# “Neuroacanthocytosis” – Overdue for a Taxonomic Update

**DOI:** 10.5334/tohm.583

**Published:** 2021-01-11

**Authors:** Ruth H. Walker, Adrian Danek

**Affiliations:** 1 James J. Peters VAMC, Bronx, Department of Neurology, Mount Sinai School of Medicine, New York City, NY, US; 2 Neurologische Klinik und Poliklinik, Ludwig-Maximilians-Universität München, Munich, Germany, DE

**Keywords:** neuroacanthocytosis, acanthocytosis, McLeod syndrome, chorea-acanthocytosis, VPS13A

## Abstract

The term “neuroacanthocytosis” (NA) is used for a spectrum of neurological disorders in which there are thorny red blood cells. While NA historically referred to disorders of lipoprotein absorption, we have promoted it as an overarching term for a group of basal ganglia disorders, with specific reference to two diseases that we defined as “core” NA syndromes. “Neuroacanthocytosis” has also been used to refer to a specific, now genetically-defined disease, otherwise known as “chorea-acanthocytosis”. These various usages have resulted in diagnostic confusion, and in a number of cases have quite likely prevented the pursuance of precise, molecular, diagnosis. Disease nomenclature is an ever-evolving field, especially in the current era of expanding genetics, and naming proposals are often far from ideal. We, however, suggest that the term “neuroacanthocytosis” should no longer be generally used and if so, only with appropriate understanding of its limitations. Further, we propose that chorea-acanthocytosis be renamed as “*VPS13A* disease” in accordance with its genetic etiology.

## INTRODUCTION

Use of the term “neuroacanthocytosis” (NA) has been fraught with taxonomic confusion over the years. This persistent confusion is predominantly due to the fact that NA has been used at various different levels of classification to refer to neurological diseases in which deformed, “spiky” erythrocytes (acanthocytes; in other contexts also referred to as spur cells [1]) can be seen. Most broadly, NA refers to: 1) disorders of lipid absorption (such as Bassen-Kornzweig disease), in which there are peripheral neuropathy and cerebellar signs and 2) basal ganglia degenerative disorders, characterized by movement disorders. The former group of disorders is now generally, and more properly, defined by their metabolic or genetic abnormalities (such as abetalipoproteinemia or *MTTP* mutations, respectively).

At a more specific level, NA, as we defined it almost 20 years ago [2], is an umbrella term referring to the second group of disorders. Among these, we designated two diseases as “core” NA syndromes, chorea-acanthocytosis (ChAc; autosomal recessive, OMIM #200150) and McLeod syndrome (X-linked recessive, OMIM #300842), grouping them with two additional disorders, Huntington’s disease-like 2 (HDL2; autosomal dominant, OMIM #606438) and pantothenate kinase-associated neurodegeneration (PKAN; autosomal recessive, OMIM #606157), in which acanthocytosis has also been reported.

At the third level, NA is also, and continues to be, used as synonymous with ChAc, to refer to patients with a specific neurodegenerative disorder due to identified bi-allelic mutations of *VPS13A*.

Originally, it was hoped that retention of the umbrella term NA would fertilize research by hinting at possibly shared pathways of various molecular mechanisms [3]. We now argue that the term ought to be replaced or at least used only with the greatest care, in order not to impede identification of a more precise diagnosis for the individual patient in the current age of “precision medicine”.

## DISCUSSION

There are a number of arguments in support of our proposal to update the current and previous taxonomy. Yamamoto and collaborators introduced the term “neuroacanthocytosis” in 1982 for “a combination of neurologic disorder and acanthocytosis” or “neurologic disease and acanthocytes occurring together” under which they included cases with lipid abnormalities. Their presentation of two siblings [4], however, was focused on “familial neuroacanthocytosis with normal serum lipoprotein levels”, small case series of which were being reported from Japan [5]. In 1985 Jankovic in particular advocated for the use of “neuroacanthocytosis” rather than of “choreoacanthocytosis” or “Levine-Critchley syndrome” to describe such cases [6], that in hindsight are characterized by the features of disease due to *VPS13A* mutations. While it was important at the time to emphasize that the presentation of the disorder could be with tourettism or bradykinesia, and not just chorea, we have concerns about the continued utility of this term. One major factor influencing our position is the continued reference in the literature to a large case series by Richard Hardie and colleagues at the National Hospital Queen Square, London, from the early 1990s [7]. While the 19 reported cases with “neuroacanthocytosis” established a landmark in the field, the paper established the erroneous and persistent impression that the patients were affected by a single disorder. As has subsequently been demonstrated with molecular studies of Hardie’s cases, in addition to patients with ChAc, the series contained an atypical female carrier with a McLeod gene (*XK*) mutation and compatible symptoms, and at least three cases with PKAN [8,9].

### THE VARIABILITY OF ACANTHOCYTOSIS

A related, common misconception concerns the role of acanthocytosis for diagnosis: the conviction still appears prevalent that neither ChAc nor McLeod syndrome are likely unless acanthocytes were seen on peripheral blood smear at least once out of three attempts. Acanthocytosis is notoriously difficult to identify [10,11]. In the only systematic blinded study of peripheral acanthocytosis [10], Storch and colleagues incubated samples of peripheral blood with heparinized normal saline (1:1) for 30–120 minutes at room temperature on a shaker, and examined wet, unfixed smears using phase-contrast microscopy. While this method has become the most widely-accepted for determination of acanthocytes, the authors note that this method does not distinguish between acanthocytes and echinocytes, and they did not validate the presence of acanthocytes using the most definitive methodology, which is examination of glutaraldehyde-fixed erythrocytes using electron microscopy. Elevation of creatine phosphokinase (CK) in our experience [12] is a much more helpful indicator for the likelihood of either ChAc or McLeod syndrome, with normal CK levels arguing against these diagnoses.

### LEVINE-CRITCHLEY SYNDROME

Historically, patients with signs and symptoms consistent with NA were diagnosed with “Levine-Critchley syndrome”. The original family in Kentucky studied by Edmund Critchley [13] has been confirmed as carrying mutations of *VPS13A* [14]. We have, however, been unable to identify any surviving relatives of Levine’s family for genetic confirmation, and various features of this pedigree [15,16], specifically the autosomal dominant mode of inheritance and atypical neurological signs, raise the concern that their symptoms were due to a different disease, rendering this term unequivocally obsolete.

### MCLEOD SYNDROME

Although often clinically resembling ChAc in its neurological presentation, McLeod syndrome is a genetically distinct disorder [17], named for the original proband, Hugh McLeod, who was determined to have the specific erythrocyte antigen phenotype while a Harvard dental student in the 1960s [18]. This antigen phenotype has implications for transfusion medicine [19]; it is also important to make this diagnosis as patients may benefit from regular screening to evaluate for cardiac involvement which can be a significant cause of morbidity and mortality, and may even necessitate cardiac transplantation [20].

### HUNTINGTON’S DISEASE-LIKE 2

Anderson et al. systematically reviewed the features of HDL-2 and incorporated the findings of acanthocytosis that we had made in four patients [21]. However, in a subsequent systematic, blinded study comparing blood smears from a cohort of 12 HDL2 patients with smears from Huntington’s disease (HD) patients and controls [22] they failed to confirm our observations. In light of this, we recommend that HDL2 no longer be considered as an NA syndrome. There is no clear explanation for our original observations, which were not only documented in three members from one family [23] but also in a patient from a different clinical center in addition to our own [24]. One possible explanation could be nutritional compromise [1]. However, our patients were not dramatically underweight at the time of examination, and weight loss is common to the majority of patients with HDL2, and indeed, HD.

### PANTOTHENATE KINASE-ASSOCIATED NEURODEGENERATION

PKAN is widely recognized as the prototypic neurodegeneration with brain iron accumulation (NBIA) disorder, not least because of its test-friendly “eye-of-the-tiger” sign on brain MRI. Acanthocytosis is documented in about 8% of patients with PKAN [25], possibly caused by dysfunction of lipid synthesis due to the enzyme defect. With genetic testing available, PKAN has transpired to be the diagnosis in the two cases that had entered the literature under the “HARP” acronym (hypoprebetalipoproteinemia, acanthocytosis, retinitis pigmentosa, pallidal degeneration) [26,27,28,29], in spite of general doubts as to the utility and even meaning of “hypoprebetalipoproteinemia” [30]. As the clinical and radiological features of PKAN are at this point well-defined and well-recognized, we feel that it does not serve any useful purpose to include PKAN as an NA syndrome.

### OTHER MOVEMENT DISORDERS WITH ACANTHOCYTOSIS

“Familial acanthocytosis with paroxysmal exertion-induced dyskinesias and epilepsy” [31] was later found to be caused by a *GLUT1* mutation [32]; acanthocytes have additionally been reported in a variety of neurogenetic disorders, e.g. with mutations of *ELAC2* [33] or in aceruloplasminemia [34]. Such findings are intriguing, but terming these disorders “neuroacanthocytosis” only serves to perpetuate and increase the taxonomic confusion. The latter is evident from the ongoing use of “neuroacanthocytosis” as a definitive diagnosis for cases with a movement disorder and acanthocytosis in spite of absent genetic or protein-based analyses [35,36]. We suspect that this indicates an insufficient understanding of the clinical constellations and the diagnostic framework of these disorders [37]. It is, however, critical that appropriate genetic diagnoses be made as molecular therapies become available.

### VPS13A DISEASE/CHOREA-ACANTHOCYTOSIS

In patients with neurodegenerative disease due to bi-allelic mutations of *VPS13A* (“chorea-acanthocytosis”), neither chorea nor acanthocytes are obligatory or invariable features. This has been illustrated by cases with neither of these features, in whom the diagnosis was confirmed by impaired expression of the *VPS13A* gene product, chorein, on Western blot^1^ [38,39]. We have previously supported the use of “chorea-acanthocytosis” as a disease designation for this disorder in spite of our own experience with various combinations of cognitive, behavioral, epileptic, neuromuscular, and movement disorders in this multi-faceted condition [12]. Going forward, however, and inspired by genetics-based taxonomy we now propose that the condition be renamed as “*VPS13A* disease”; while this remains an unwieldy term and far from ideal, we wish to avoid the equally unwieldy and incomplete shorthand term (CHOR-VPS13A) suggested by the Nomenclature Task Force of the Movement Disorders Society [40].

## CONCLUSION

If used as an overarching term referring only to *VPS13A* disease and McLeod syndrome, “neuroacanthocytosis” might retain some utility. Apart from this special situation, however, we strongly recommend to authors, editors and reviewers that the term is employed very judiciously, and that close attention should be paid to determine whether “neuroacanthocytosis” was perhaps being used as an ambitious, yet empty, diagnostic cover for incompletely worked-up clinical presentations.

We are aware that changing disease nomenclature is typically an arduous and lengthy process, and can result in increasing rather than reducing confusion, at least temporarily. We hope, however, that ultimately our current proposal for naming *VPS13A* disease with its definitional genetic cause will have a positive outcome for clinicians, researchers, and of course, affected patients and their care-partners.

## Footnotes

The free diagnostic Western blot test for chorein is sponsored by the NA patient organization, *www.naadvovacy.org*. For instructions see: *www.euro-hd.net/edit/na/network/docs/chorein_wb_info.pdf*.
